# Cost‐effectiveness analysis of silver diamine fluoride to divert dental general anaesthesia compared to standard care

**DOI:** 10.1111/adj.12936

**Published:** 2022-09-29

**Authors:** TM Nguyen, U Tonmukayakul, M Hall, H Calache

**Affiliations:** ^1^ Deakin Health Economics, Institute for Health Transformation Deakin University Burwood Victoria Australia; ^2^ Dental Health Services Victoria Carlton Victoria Australia; ^3^ Public Health and Preventive Medicine Monash University Melbourne Victoria Australia; ^4^ La Trobe Rural Health School La Trobe University Bendigo Victoria Australia

**Keywords:** Children, cost‐effectiveness analysis, dental caries, general anaesthesia, silver fluoride

## Abstract

**Background:**

The aim is to perform a model‐based cost‐effectiveness analysis of a silver diamine fluoride (SDF) protocol intervention to divert dental general anaesthesia (DGA) among Victorian children aged 2–10 years.

**Methods:**

Data inputs were based on an Australian single‐cohort 2017/18 study. Intervention costs for standard care were derived from two subgroups of children: (1) children who received standard care without DGA, and (2) children who received standard care with DGA. Two scenarios were modelled due to limited post‐follow‐up data: (1) children receiving SDF had standard care without DGA (base‐case scenario), and (2) children receiving SDF did not receive standard care without DGA (alternative scenario). A simple decision‐tree model with probabilistic sensitivity analysis (PSA) estimated the incremental costs per diverted DGA.

**Results:**

The probability of children requiring specialist referral and offered SDF, but the primary carer opted for DGA is 0.124 (SD 0.034), and the probability of children requiring DGA in standard care is 0.346 (SD 0.036). For both the base‐case and alternative scenario, the incremental cost‐effectiveness ratio outcome is dominant and their cost‐effectiveness being either 74.8% or 100% respectively.

**Conclusions:**

The SDF protocol intervention is cost‐effective dental caries management option for young children where referral for DGA is considered. © 2022 Australian Dental Association.

**Abbreviations and acronyms**
ADA: Australian Dental Association
DGA: dental general anaesthesia
DWAU: dental weighted activity unit
ECC: early childhood caries
NCDs: non‐communicable chronic diseases
PPHs: potentially preventable hospitalizations
PSA: probabilistic sensitivity analysis
SDF: silver diamine fluoride

## INTRODUCTION

Early childhood caries (ECC) is one of the most prevalent and preventable non‐communicable chronic diseases (NCDs), with a global estimate of 48% (95% CI 42; 53).^1^ It is defined as at least one decayed (non‐cavitated or cavitated), missing or filled (due to caries) surfaces of any deciduous tooth for a child under 6 years of age.^2^ ECC is a biofilm‐mediated, sugar‐driven, multifactorial, dynamic disease caused by the unfavourable homeostasis of demineralization and remineralization of the dental hard tissues.^2^

Preservation of the primary dentition is essential to a child's well‐being and development.^2^ In Australia, one in three (34%) preschool children aged 5–6 years have a history of caries experience in the deciduous dentition, of which 26% of children in the same age group have untreated ECC.^3^ Among older children, 27% of children aged 5–10 years and 11% of children aged 6–15 years have untreated dental caries in the deciduous and permanent dentition respectively.^3^ Pre‐school and primary school children of lower socioeconomic backgrounds have a greater risk for developing ECC and experience greater severity.^3^, ^4^

The burden on the healthcare system due to untreated ECC and dental caries among children is significant. Over 90% of potentially preventable hospitalizations (PPHs) for children aged 0–4 years and 5–9 years in Victoria, Australia, had the principal diagnosis of dental caries.^5^ The rate of PPHs are the highest for both these age groups compared to all other age groups in Australia, at 4.9 separations per 1000 of PPHs and 9.5 separations per 1000 of PPHs for the 0–4 year and 5–9 year age groups respectively.^6^ Dental conditions are the third most common cause of PPHs for acute conditions, and comprises 10% of the total number of PPHs in Australia.^7^ The cost allocation for hospital funding is estimated to be AUD $16 987 per case according to the Australian Refined Diagnosis Related Groups (AR‐DRG) Version 10 (D40Z—Dental Extractions and Restorations).^8^, ^9^

ECC management can be impractical on dental chair, largely due to the limitations of patient co‐operation.^10^ Other factors include the confidence of the dental practitioners,^11^ primary carer preferences,^12^ and may require multiple visits. For these reasons, dental general anaesthesia (DGA) has been a traditional management option to provide comprehensive dental caries treatment, particularly when multiple carious teeth needed be treated in one visit. However, the DGA may not necessarily reduce dental anxiety,^13^, ^14^, ^15^, ^16^ and can result in children receiving less regular preventive oral healthcare^15^, ^17^ given the severe stages of dental caries has been addressed. Alternative interventions of DGA include anxiolysis and conscious sedation,^13^, ^18^, ^19^ and the minimally invasive dentistry (MID) techniques for managing moderate and severe stages of dental caries. The MID approach includes atraumatic restorative treatments (ART),^20^, ^21^, ^22^, ^23^ with/without a combination with stainless steel Hall crown technique,^24^, ^25^, ^26^, ^27^ and/or application of silver diamine fluoride (SDF).^28^, ^29^, ^30^, ^31^ Currently, the Australian fluoride guidelines,^32^ states that ‘silver diamine fluoride or silver fluoride might be used for people with caries in situations where traditional treatment approaches to caries management might not be possible’. However, SDF has the potential to enhance dental caries management non‐invasively in routine practice, resulting in preserving tooth structure and is less costly than surgical interventions if cost is a financial constraint by the client.

Regular application of SDF has demonstrated the potential in arresting dental caries.^33^, ^34^, ^35^, ^36^ The advantages of SDF in comparison to DGA are its ease of application, patient acceptability, shorter treatment time and costs. In 2017/18, an Australian‐based prospective single‐cohort study incorporated SDF alongside a comprehensive oral health education intervention (the SDF protocol intervention). The oral health education included the promotion of twice daily toothbrushing with fluoride toothpaste and dietary modification. The SDF protocol intervention was effective in reducing up to 88% of dental referrals for DGA to the Royal Dental Hospital of Melbourne (RDHM) Victoria, in two selected Victorian community dental agencies.^37^ There was also a significant improvement to the quality of life, elicited from the Early Childhood Oral Health Impact Scale (ECOHIS),^38^ among children who received the SDF protocol intervention.^37^

While evidence demonstrating the effectiveness of the SDF has been well documented, its cost‐effectiveness and implications for the use of SDF protocol intervention in practice remain unknown. The economic benefit of the SDF can inform investment decisions for resource prioritization. Therefore, the specific aim of this study is to perform a cost‐effectiveness analysis (CEA), from the healthcare perspective, of the SDF protocol intervention for Victorian children aged 2–10 years compared to standard care to divert DGA.

## MATERIALS AND METHODS

**Fig. 1:**
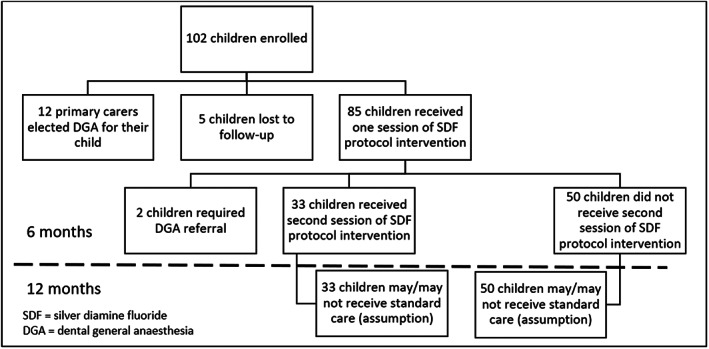
The SDF protocol intervention recruitment flow chart.

This model‐based CEA utilized information extracted from a published Victorian‐based SDF protocol intervention paper and a dental services audit from the Royal Dental Hospital Melbourne (RDHM). The details of the recruitment and outcome are published elsewhere (Fig. 1).^37^ The SDF protocol intervention prospectively observed a cohort of 102 Victorian children aged 2–10 years for 6 months. Participants were recruited in November 2017 from two selected community dental agencies in Victoria. Of these, 12 parents of children elected for DGA, 85 children completed the 6‐month follow‐up period, and five children were lost to follow‐up and excluded from the analysis.^37^ At the end of the study period where the SDF protocol intervention was offered, there was an 88% reduction in the initial referral for treatment under DGA. While the actual SDF study employed pre‐post methodology, we chose the standard care, which includes two subgroups of children: (1) those who receive standard care without DGA, and (2) those who received dental treatment under DGA, as the comparator.

The costs of the SDF protocol intervention were not collected in the study. So, treatment costs were derived from a multiplication of Dental Weighted Activity Unit (DWUA) of each item codes and the cost allocation of AUD$439.65 per DWUA. The item codes were retrieved from the dental services audit, and matched according to the Australian Dental Association dental item code descriptions.^39^ We conservatively assumed that in one visit of the SDF protocol intervention, each child received one dental visit inclusive of a dental consultation, dietary analysis and advice, oral hygiene instructions and each carious lesion received one service of a topical cariostatic agent. We assumed each child required four topical cariostatic agent applications because the mean active carious lesions in the SDF protocol intervention was 3.48 per child.^37^

Based on the published SDF protocol intervention study, 33 children required a second session of the SDF protocol at the 6‐month follow‐up due to residual active dental caries, and two children required referral for DGA.^37^ Given there was limited follow‐up beyond 6 months, we simulated two scenarios based on the primary purpose of SDF is to stabilize dental caries until children are mature enough to receive standard care. They are (1) children who received SDF had standard care without DGA in the first year (base‐case scenario), and (2) children who received SDF did not have standard care without DGA in the first year (alternative scenario). The costs of DGA for standard care were applied for children who required referral for DGA after receiving one session of the SDF. Thus, the CEA was performed over a 1‐year time horizon (Fig. 1).

Treatment costs of the standard care were estimated from an analysis of dental services provided for children aged 2–10 years old who were referred to the paediatric department at RDHM in 2018. Dental records in 2018 were selected to avoid the disruption in service delivery during the Covid‐19 restrictions to dental services during 2020–2022. Two subgroups of children aged 2–10 years old were used to inform the costs for standard care. The first cohort were children who were referred to RDHM received ‘usual care’ dental treatment without DGA. Of this group, the probability of children who were put on the DGA waiting list was identified and assumed to received DGA within 1 year. The second cohort was children who received DGA at the RDHM in 2018. Costs of dental treatments for standard care were estimated using the similar approach for SDF protocol intervention.

Statistical analysis was performed using Stata IC Version 12 (Statacorp™). Treatment costs were reported in Australian dollars in 2018 price. Given time horizon of the model is 1 year, discounting was not applied. Extrapolation of the effectiveness was not attempted due to limited information of treatment consequences beyond 1 year.

**Fig. 2:**
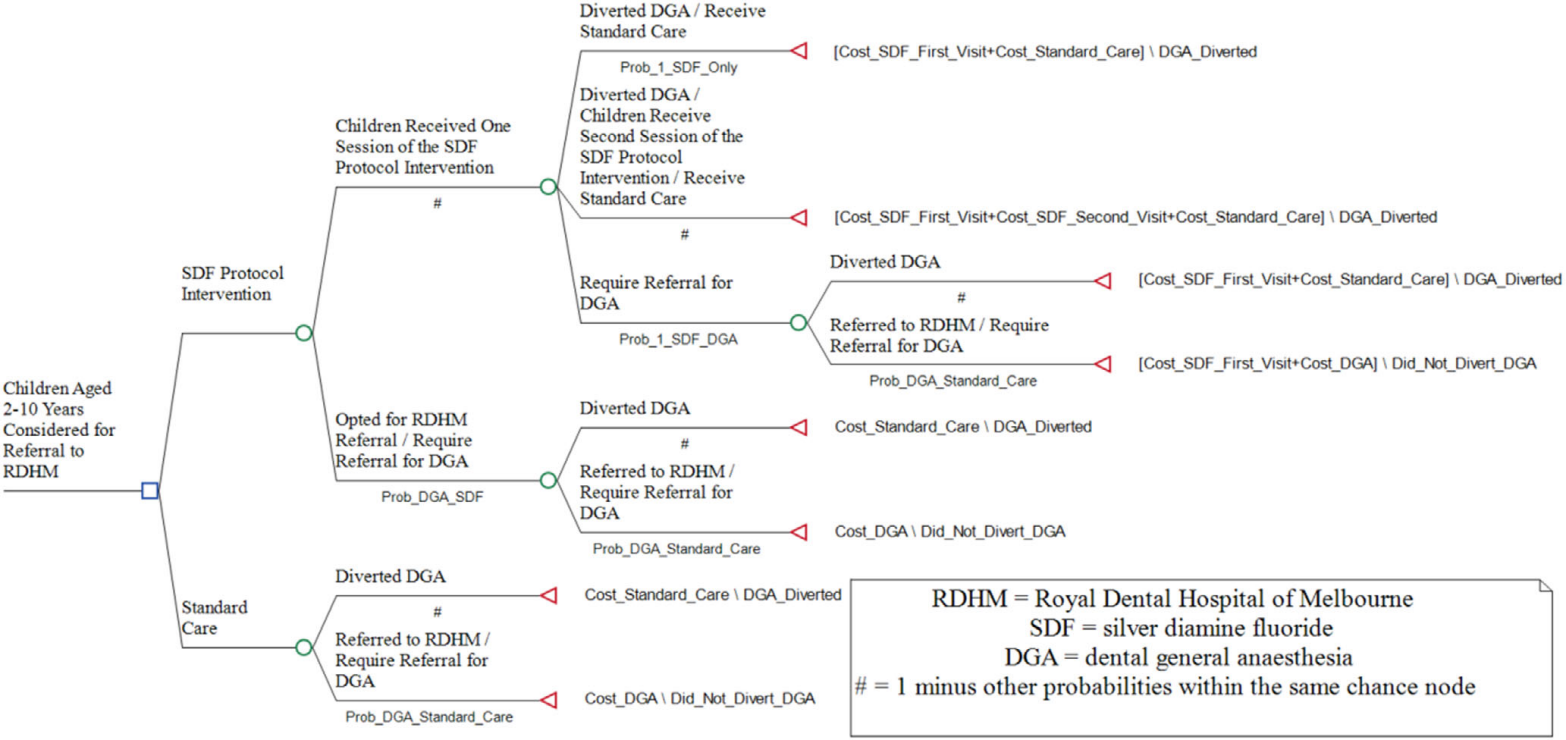
The simple decision‐tree model for the CEA of the SDF protocol intervention under the base‐case scenario as an example.

A simple decision‐tree model was constructed for the CEA (in Fig. 2) using Treeage Pro 2022 (TreeAge Software, LLC.). The incremental costs per diverted DGA were estimated, also known as the Incremental Cost‐Effectiveness Ratio (ICER).

**Fig. 3:**
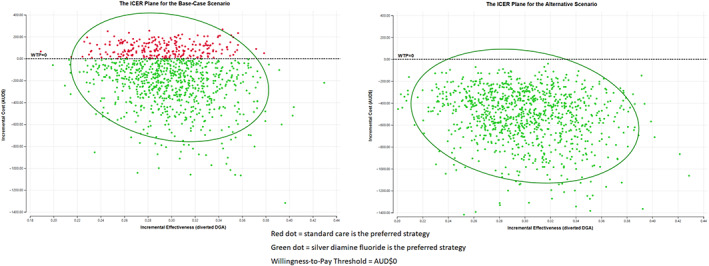
The results of the ICER plane under PSA for the base‐case and alternative scenarios.

Probabilistic sensitivity analysis (PSA) was performed using Monte Carlo simulation with 1000 cycles to simulate 1000 trials by using random sampling of the parameter inputs for each variable distribution. This is illustrated in the cost‐effectiveness plane (Fig. 3), which is used to generate the mean ICER value and corresponding uncertainty intervals. The PSA results were presented on cost‐effectiveness plane presenting the differences in costs and effectiveness of the intervention compared to the comparator. PSA is a technique to quantify the level of confidence of the analysis output by incorporating uncertainty in the model input variables.

This cost‐effectiveness study was reviewed and obtained ethics approval from [removed for blind peer review].

## RESULTS

The mean costs for the first session of the SDF protocol intervention are AUD$190.81 and the mean costs for the second session of the SDF protocol intervention are AUD$131.24 (cost variation is based on less active carious lesions requiring reapplication of SDF). The profile of dental services under the standard care is presented in Table 1.

**Table 1:** The dental service profile under standard care inclusive of two subgroups of children aged 2–10 years old who received dental services with or without DGA in 2018 at RDHM

Type of dental service^a^	Dental services provided for children who received standard care without DGA	Dental services provided for children who received standard care with DGA
	Number of Services per 100 Individuals (N = 153)	Number of Services per 100 Individuals (N = 1786)
Diagnostic services		
Oral examination	64.7	62.4
Consultation	47.7	98.7
Intra‐oral Radiographs	108.5	111.5
Preventive services		
Plaque and Calculus removal	54.9	47.3
Topical fluoride	54.2	43.8
Fissure sealant	115.7	118.0
Dietary advice	30.1	0.1
Oral hygiene Instruction	69.9	1.0
Oral surgery		
Simple extraction	15.0	395.2
Surgical extraction	0.0	2.7
Restorative services		
Adhesive anterior restorations	6.5	61.7
Adhesive posterior restorations	25.5	94.1
Stainless steel crown	8.5	132.6
General services		
Use of interpreter	13.1	28.9

^a^ Description of dental services are matched to the relevant ADA dental item codes and associated costs using the DWUA funding model as listed in Appendix 1.

The parameters inputs for the decision‐tree model are reported in Table 2. The mean costs for children receiving standard care without DGA (N = 153) are AUD$409.90 (SD 36.24). For standard care, the mean cost for children receiving one DGA session (N = 1786) is AUD$1793.23 (SD 803.45) and its probability is 0.346 (SD 0.036).

**Table 2:** Detailed parameter inputs for the CEA of the SDF protocol intervention against standard care

Parameter inputs for the economic evaluation model	Variable name in Treeage	Mean (SD)	Distribution
Probability of children where the primary carer opted for DGA	Prob_DGA_SDF	0.124 (0.034)	Beta
Probability of children receiving one session of the SDF protocol intervention and did not require DGA	Prob_1_SDF_Only	0.365 (0.052)	Beta
Probability of children receiving one session of the SDF protocol intervention and required DGA	Prob_1_SDF_DGA	0.0236 (0.016)	Beta
Probability of children in standard care required DGA	Prob_DGA_Standard_Care	0.346 (0.036)	Beta
Cost of first session of the SDF protocol intervention (AUD$)	Cost_SDF_First_Visit	190.81	‐
Cost of second session of SDF protocol intervention (AUD$)	Cost_SDF_Second_Visit	131.24	‐
Cost of standard care without DGA (AUD$)	Cost_Standard_Care	409.90 (36.24)	Gamma
Cost of standard care with DGA (AUD$)	Cost_DGA	1793.23 (803.45)	Gamma
Diverted DGA	Diverted_DGA	1	‐
Did not divert DGA	Did_Not_Divert_DGA	0	‐

Under the base case scenario, the economic evaluation resulted in a mean cost‐saving of AUD$171.01 (95% CI −185.91; −156.10) per child and had a mean effectiveness of 0.298 (95% CI 0.296; 0.301) of diverted DGA, for 1 year. The alternative scenario yielded a mean cost‐saving of AUD$518.50 (95% CI −534.00; −503.01) per child and had a mean effectiveness of 0.300 (95% CI 0.298; 0.302) of diverted DGA.

**Fig. 4:**
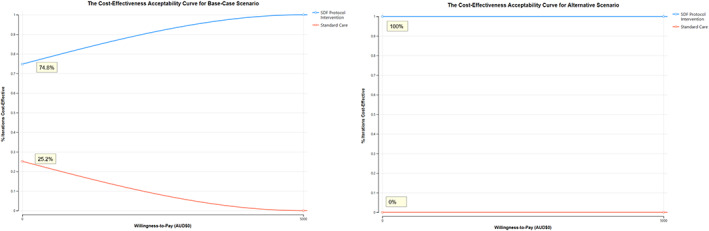
The results of the cost‐effectiveness acceptability curve for the base‐case and alternative scenarios.

The 1000 plausible incremental costs per diverted DGA values are illustrated on the ICER plane (Fig. 3) and mean ICER value is presented in Table 3. Both the base‐base and alternative scenarios had a dominant result. Under PSA when the SDF protocol intervention does not have an incremental cost or incremental effectiveness, that is the willingness‐to‐pay is equal to 0 (WTP = 0), the intervention ranged from 74.8% cost‐effective for the base‐case scenario to being 100% cost‐effective with the alternative scenario as represented in the cost‐effectiveness acceptability curve (Fig. 4).

**Table 3:** The CEA results of the SDF protocol intervention against standard care

Outcomes	Mean (SD)	95% Confidence Interval
Base case scenario		
Incremental cost (AU$)	−171.01 (7.59)	−185.91; −156.10
Incremental effectiveness (diverted DGA)	0.298 (0.001)	0.296; 0.301
Incremental cost‐effectiveness ratio	−562.57 (24.87)^a^	−611.37; −513.76
Alternative scenario		
Incremental Cost (AU$)	−518.50 (7.90)	−534.00; −503.01
Incremental Effectiveness (diverted DGA)	0.300 (0.001)	0.298; 0.302
Incremental cost‐effectiveness ratio	−1734.228 (25.89)^a^	−1785.02; −1683.43

^a^ Dominant.

## DISCUSSION

The dominant outcome when the ICER value was calculated in our study demonstrates the SDF protocol intervention saved costs and it was more effective to divert DGA compared to standard care. The yielded cost‐savings are consistent with previous research from a US simulation study demonstrating the potential cost‐savings would range between US$100 to US$350 per restorative visit,^40^ or US$201 per 3‐month period per visit,^41^ by Medicaid‐enrolled children. In another study, children aged under 6 who received SDF for dental caries management had an overall mean cost‐saving of US$292 per year, which included children who later received DGA.^42^ The average costs for DGA in our study of AUD$1793 appears comparable to previous cost estimates of AUD$1554 in Western Australia associated with PPHs due to dental conditions among children aged 2–16 years old.^43^ Our study expands on the existing literature by demonstrating the economics benefits for adopting the SDF protocol intervention from a healthcare perspective in the Australian context.

The study outcome is not surprising given there is strong evidence for the clinical effectiveness of the application of SDF for arresting dental caries.^28^ However, our study highlighted there were low levels of preventive services provided for the cohort of children referred for DGA, who are at high risk for dental caries, such as topical fluoride, dietary analysis and advice and oral hygiene instructions. A plausible reason is that these dental services may have been provided either before the referral or post‐operatively at clinical handover by referring back to the Victorian community dental agencies. The rate for the use of stainless steel crowns was greater than using adhesive restorations (such as glass ionomer cements or composite resin) under the DGA, which is preferred given its superiority in reducing the risk of major failure or pain in the long term compared to adhesive restorations.^44^ The different variations in oral surgery and restorative procedures indicate that children in standard care who were referred to RDHM included those who probably did not require DGA, and may be for other reasons. Our CEA model captured the outcomes that would have occurred in the real world. A future robust study on the SDF protocol intervention that includes economic evaluation running alongside the trial would validate our results.

Whilst not explicitly considered in the Australian SDF protocol intervention study,^37^ there may have been additional benefits that are worthwhile discussing. Children who were exposed to the SDF protocol intervention are more likely to have more regular appointments as part of the dental caries management protocol, which can provide more opportunities to reinforce preventive oral health messages. In the SDF protocol intervention study, there were anecdotal evidence reported by mothers who were ‘delighted’ and appreciated the dental problem was resolved without requiring DGA. A preventive approach using SDF is preferred by primary carers over DGA despite tooth staining concerns associated with the use of SDF.^45^ The aesthetics concerns of tooth staining could be alleviated by using potassium iodide after the application of SDF.^46^ While the SDF protocol intervention may not necessarily provide definitive dental treatment, ‘desensitization’ dental visits using SDF can delay surgical dental treatment needs and reduce the likelihood of dental caries deterioration until children a mature to accept standard care.

More regular dental appointments via the SDF protocol intervention are consistent with the US study, where children who received SDF applications according to the American Academy of Pediatric Dentistry (AAPD) guidelines,^47^ had significantly more dental visits but less restorative visits and overall treatment costs.^42^ A key difference between the Australian SDF protocol intervention study and the AAPD guidelines, is the shorter time interval for the SDF application using the AAPD guidelines at 2 weeks and 6 months thereafter. It should be noted that the AAPD guidelines were not available at the time when the Australian SDF protocol intervention trial was approved. A shorter interval between the first and second application of SDF could generate larger cost‐savings if the clinical effectiveness of the SDF protocol intervention at 54% increase between 65% and 91%, which have been reported in previous studies.^28^

Given the intervention is cost‐saving and more effective to divert DGA, the broader adoption of the SDF protocol intervention would release healthcare resources. This can hasten access to oral healthcare for children where the use of SDF would have negligible impact on DGA outcomes such as acute dental trauma or established symptomatic carious lesions including pain, swelling and active dental infections (abscess). Alternatively, the freed resources can be redirected to fund and provide other healthcare services. Greater impact for minimizing expenditure on healthcare resources would occur for the SDF protocol intervention if it were to be implemented in routine practice at the local Victorian community dental agency, except where dental extractions indicated. Lastly, any outcome to divert DGA would minimize the risk of mortality, which is estimated to be 3 per 1 million persons for dental treatment under DGA.^48^

### Limitations

The results of this economic evaluation should be interpreted with caution given the data inputs were not derived from a randomized controlled trial, the time horizon is a short duration (1 year), and the sample size included children up to 10 years old. It is possible that children who received the SDF protocol intervention may only delay their inevitable dental caries management trajectory towards DGA after 1 year. Another limitation related to the cost component is the unknown direct dental treatment costs for the SDF protocol intervention prior to the referral to RDHM and any subsequent costs after the 6‐month follow‐up. A longer follow‐up period is needed to determine whether the underestimate of the dental treatment costs for the SDF protocol intervention would significantly affect the probability for cost‐effectiveness.

Other cost considerations not included in this CEA are the potential cost consequences external to the health service sector, that is, the societal perspective. Standard care provided under DGA can minimize the opportunity costs lost by the primary carer due to taking time off work and the travel costs to and from the dental clinic. These societal costs could be higher for SDF protocol intervention given the greater likelihood to attend multiple appointments for review and re‐application of SDF. The exclusion of the societal costs may influence the CEA results. At an individual level, the societal costs could vary dramatically, that is, primary carers of children living in rural and remote areas, where regular access to preventive services is scarce, may opt for DGA if they cannot accept the societal costs.

## CONCLUSION

Our CEA study provides evidence to strengthen the statement regarding the use of SDF by the Australian fluoride guidelines to promote its broader adoption amongst dental practitioners. The SDF protocol intervention is cost‐effective with high degree of certainty. It is recommended that the SDF protocol intervention is the preferred option advocated by dental practitioners where referral for DGA is considered for dental caries management. Future research should explore the potential longer‐term consequences for incorporating the SDF protocol intervention over a longer‐time horizon, and whether its cost‐effectiveness is maintainable.

## Ethics Approval

Deakin University Human Research Ethics Committee (ID 2021‐431).

## APPENDIX 1 The descriptions of the ADA dental item codes being matched against the equivalent DWAU value

**Table:**

Type of dental service	ADA dental item code	Description of dental service	DWAU Value
Diagnostic services			
Oral examination	011	Comprehensive oral examination	0.09
	012	Periodic oral examination	0.08
	013	Oral Examination – limited	0.05
Consultation	014	Consultation	0.11
	016	Consultation – extended (30 minutes or more)	0.18
Intra‐oral radiographs	022	Intraoral periapical or bitewing radiograph – per exposure	0.06
	024	Intraoral periapical or bitewing radiograph – each subsequent exposure (same day)	0.06
	025	Intraoral radiograph – occlusal, maxillary or mandibular – per exposure	0.11
Preventive services			
Plaque and Calculus removal	111	Removal of plaque and/or stain	0.09
	114	Removal of calculus – first visit	0.15
	115	Removal of calculus – subsequent visit	0.10
Topical fluoride	121	Topical application of remineralization and/or cariostatic agents, one treatment	0.06
	123	Concentrated remineralization and/or cariostatic agents, application – single tooth	0.05
Fissure sealant	161	Fissure and/or tooth surface sealing – per tooth (first 4 services on a day)	0.08
	162	Fissure and/or tooth surface sealing – per tooth (after 4 occasions of 161 on a day)	0.08
Dietary advice	131	Dietary analysis and advice	0.06
Oral hygiene instruction	141	Oral hygiene instruction	0.09
Oral Surgery			
Simple extraction	311	Removal of a tooth or part(s) thereof	0.23
	314	Sectional removal of a tooth or part(s) thereof	0.29
	316	Removal of additional tooth or part(s) thereof – same quadrant per day	0.23
Surgical extraction	322	Surgical removal of tooth/fragment not requiring removal of bone or tooth division	0.37
	323	Surgical removal of tooth/fragment requiring removal of bone	0.42
	324	Surgical removal of tooth/fragment requiring both removal of bone and tooth division	0.57
	326	Additional surgical removal of tooth/fragment	0.42
Restorative services			
Adhesive anterior restorations	521	Adhesive restoration – one surface – anterior tooth – direct	0.2
	522	Adhesive restoration – two surface – anterior tooth – direct	0.24
	523	Adhesive restoration – three surfaces – anterior tooth – direct	0.29
	524	Adhesive restoration – four surfaces – anterior tooth – direct	0.33
	525	Adhesive restoration – five surfaces – anterior tooth – direct	0.39
Adhesive posterior restorations	511	Metallic restoration – one surface – direct	0.18
	512	Metallic restoration – two surfaces – direct	0.22
	513	Metallic restoration – three surfaces – direct	0.26
	514	Metallic restoration – four surfaces – direct	0.3
	515	Metallic restoration – five surfaces – direct	0.34
	531	Composite resin restoration – one surface – posterior tooth – direct	0.21
	532	Composite resin restoration – two surface – posterior tooth – direct	0.27
	533	Composite resin restoration – three surfaces – posterior tooth – direct	0.32
	534	Composite resin restoration – four surfaces – posterior tooth – direct	0.36
	535	Composite resin restoration – five surfaces – posterior tooth – direct	0.42
Stainless steel crown	586	Crown metallic – with tooth preparation – preformed	0.44
	587	Crown metallic – minimal tooth preparation – preformed (Hall crown)	0.44
	588	Crown – tooth coloured – preformed	0.44
General services			
Use of interpreter	935	Interpreter	0
